# Additive Manufacturing of Patient-Customizable Scaffolds for Tubular Tissues Using the Melt-Drawing Method

**DOI:** 10.3390/ma9110893

**Published:** 2016-11-03

**Authors:** Yu Jun Tan, Xipeng Tan, Wai Yee Yeong, Shu Beng Tor

**Affiliations:** Singapore Centre for 3D Printing, School of Mechanical & Aerospace Engineering, Nanyang Technological University, 50 Nanyang Avenue, Singapore 639798, Singapore; xptan1985@gmail.com (X.T.); msbtor@ntu.edu.sg (S.B.T.)

**Keywords:** tissue engineering, scaffold, additive manufacturing, tubular tissues, melt-drawing

## Abstract

Polymeric fibrous scaffolds for guiding cell growth are designed to be potentially used for the tissue engineering (TE) of tubular organs including esophagi, blood vessels, tracheas, etc. Tubular scaffolds were fabricated via melt-drawing of highly elastic poly(l-lactide-co-ε-caprolactone) (PLC) fibers layer-by-layer on a cylindrical mandrel. The diameter and length of the scaffolds are customizable via 3D printing of the mandrel. Thickness of the scaffolds was varied by changing the number of layers of the melt-drawing process. The morphology and tensile properties of the PLC fibers were investigated. The fibers were highly aligned with a uniform diameter. Their diameters and tensile properties were tunable by varying the melt-drawing speeds. These tailorable topographies and tensile properties show that the additive-based scaffold fabrication technique is customizable at the micro- and macro-scale for different tubular tissues. The merits of these scaffolds in TE were further shown by the finding that myoblast and fibroblast cells seeded onto the scaffolds in vitro showed appropriate cell proliferation and distribution. Human mesenchymal stem cells (hMSCs) differentiated to smooth muscle lineage on the microfibrous scaffolds in the absence of soluble induction factors, showing cellular shape modulation and scaffold elasticity may encourage the myogenic differentiation of stem cells.

## 1. Introduction

Tissue engineering (TE) is an approach to alleviate the vast shortage of donor tissue [1]. It would potentially offer ready-to-use replacements with structural integrity and normal function [2]. These constructs consist of cells or biodegradable biomaterials, or both, allowing tissues to be organized appropriately in three dimensions, and can be used to replace the excised tissues [3]. TE scaffolds are biocompatible templates for temporary structural support which provide guidance for tissue development. The scaffolds should form the desired structures for the intended tissue, with appropriate mechanical properties to withstand mechanical stresses in vivo [4]. TE of the tubular organs, e.g., the blood vessels, gastrointestinal tracts, and trachea, is attracting attention [5,6,7,8]. A large number of surgeries are performed on tubular organs due to diseases such as carcinoma [2] or child birth defects [7], which usually require surgical gap bridging meant to restore the functions of organs [9]. TE of tubular organs represents a promising approach to regeneration.

**Figure 1:**
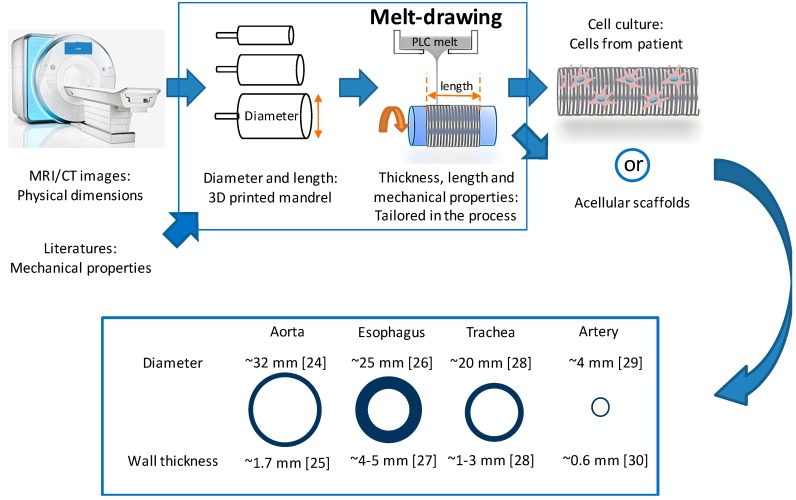
Schematic illustration of dimension and properties customization for scaffolds for tubular tissues [24,25,26,27,28,29,30] by using melt-drawing.

Fiber scaffolds have been fabricated using methods such as electrospinning [10]. However, this method requires high-voltage equipment and is sensitive to environmental control. In this work, we propose a new melt-drawing method which requires only bench-top equipment and is less sensitive to external environmental effects. Melt-drawing of fiber can be used to fabricate polymeric tubular scaffolds in one step in a layer-by-layer manner based on the concept of additive manufacturing (AM) [11,12]. AM has been demonstrated as a viable technique in 3D printing of biocompatible materials such as metals (e.g., titanium [13,14] and stainless steel [15]), ceramics [16] and polymers [17] for biomedical applications. Notable applications are implants [18] and scaffolds [19] for TE. The emerging technology of bioprinting [20,21] further enables the development of tissue and organs using AM-based methods. Based on a similar premise of layered fabrication, we proposed a new method of additively fabricating tubular scaffolds for TE. This novel method was realized using a table-top device built in-house [22]. This AM-based technique for scaffolds offers a number of advantages over other TE methods. One of these advantages is its capability to produce highly aligned fibers, whereby the fiber alignment was shown to provide the 3-dimensional (3D) alignment of cells [23]. Another advantage is the topographical control, which can be customized to achieve the required properties of a scaffold as illustrated in Figure 1. The fabrication process together with the biomaterials chosen can achieve the required mechanical properties of scaffolds. Scaffolds’ size and shape personalization are accomplished easily. Another distinct benefit of using this technique is that no undesirable toxic solvent or chemical is used. Structures of the tubular scaffolds can be formed reproducibly in one step without subsequent knitting or porogen removal. Its AM nature implies that minimal waste is produced during the fabrication. It is a green and cost-effective scaffold fabrication technique. 

TE scaffolds can be made of naturally occurring biomaterials, e.g., collagen [31], alginate [8] and gelatin [32], or synthetic biomaterials, e.g., polylactides (PLA) [33], polycaprolactones (PCL) [34,35], and polyglycolides (PGA) [36]. The selection of biomaterials usually depends on the biomechanical properties needed to match to the implantation site and also the desired degradation time frame [37]. Among these biomaterials, copolymers of poly(l-lactide) (PLLA) and poly(ε-caprolactone) (PCL), i.e., poly(l-lactide-co-ε-caprolactone) (PLC), have been used widely in the TE of elastic tissues [38,39]. PLC combines the advantage of the soft PCL with the strong and hydrophilic PLLA, most of which have rubber-like elasticity and shape memory properties [40]. Since most of the tubular organs exhibit certain elasticity for fluid and/or food bolus flow, elastic PLC is suitable to resemble them. PLC was used in our previous papers [11,12] for esophageal TE and it was shown to have tensile properties comparable to the native esophagus.

In this work, microfibrous tubular scaffolds were fabricated by melt-drawing PLC for tubular organ TE, in which the fabrication technique shows a great potential in producing the size- and properties-customized scaffolds. The in vitro biocompatibility is studied to further verify that this scaffold is suitable for cell growth and alignment. 

## 2. Results and Discussion

### 2.1. Fabrication of Customisable PLC Tubular Scaffolds

**Figure 2:**
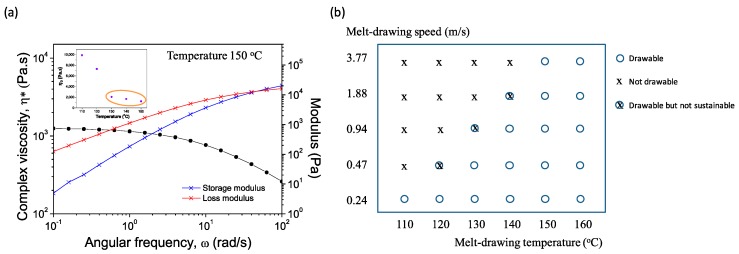
(**a**) Dynamic frequency sweep test at 150 °C. Inset illustrates the zero-shear viscosities at different temperatures; (**b**) Melt-drawing ability analysis of PLC at different melt temperatures and melt-drawing speeds.

The study of rheological properties of polymer melts is important in determining the materials’ processability. Figure 2a shows the frequency dependencies of storage modulus (G′), loss modulus (G″), and complex viscosity (η*) for PLC at 150 °C. Relationship of zero-shear viscosities of PLC melt at different temperatures are shown in the inset. PLC melts at 110 °C [12] with a viscosity of 9889 Pa·s. It starts to flow when the temperature reaches 130 °C. Figure 2b illustrates the melt-drawing ability of PLC at different temperatures and melt-drawing speeds. Here we choose the melt-drawing temperature of 150 °C for the best drawing ability of PLC, while having the least chance to degrade the polymer during processing. PLC maintains its polymeric properties and was not thermally degraded after the melt-drawing process. M_n_ and PDI of the pristine PLC measured by GPC were 13,121 and 4.18, respectively; while M_n_ and PDI of melt-drawn PLC (V_3.77_) were 15,588 and 3.82, respectively. The results indicate that the PLC chains were not broken during fabrication process, so it is expected to retain its original material properties. 

**Figure 3:**
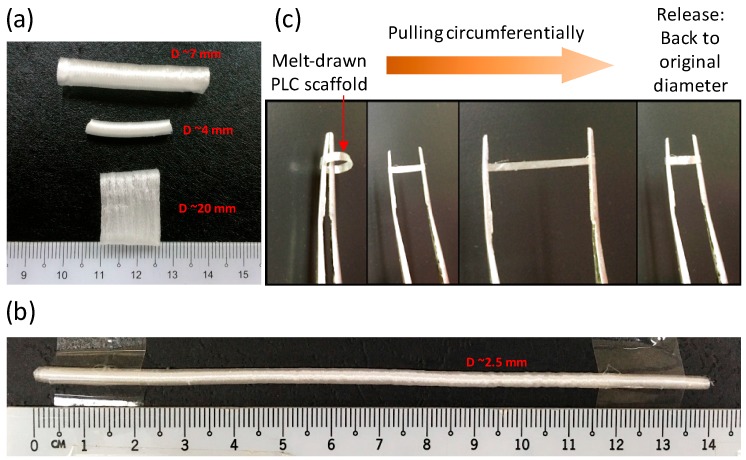
(**a**,**b**) Pictures of PLC scaffolds with different dimensions; (**c**) Illustration of elasticity of PLC scaffold in the radial direction.

Tubular scaffolds are formed in one step due to the viscoelasticity of the PLC at room temperature. Polymer fibers are merged together during melt-drawing on the tubular mandrels, thus forming tubular scaffolds. As a comparison, melt-drawn PCL in previous works were not able to form tubes [22,41]. Instead, loose PCL fibers were melt-drawn. The fabricated tubular scaffolds can be customized at different hierarchal levels, from the overall tube diameter and length, to tubular wall thickness and also the micro-fiber dimensions. Figure 3a,b shows the PLC tubular scaffolds with different overall diameter dimensions, which were fabricated using the mandrels with different dimensions. The wall thicknesses of the scaffolds were varied by altering the number of layers of the PLC melt-drawn on the mandrel. It shows the feasibility of producing a macroscale customizable scaffold for different tubular TE applications. Some tubular organs are large, such as the human aorta, which has a diameter of ~32 mm [24] with wall thickness of ~1.7 mm [25], and the esophagus, which has an average diameter ranging from ~20 to 30 mm [26] with wall thickness of ~4 to 5 mm [27]. For small diameter blood vessels, the lumen diameter can be ~0.1 to 6.0 mm with wall thickness of ~0.3 to 0.8 mm [30]. The dimensions of these tubular organs vary from person to person [24,25,29], e.g., age and gender affect the dimensions of the tubular organ significantly [24]. Of note is that it was challenging to engineer long tubes with small diameter (<6 mm) for vascular replacement. Here the PLC scaffold with diameter of ~2.5 mm, wall thickness of ~0.25 mm, and length of ~14 mm as illustrated in Figure 3b can be fabricated using melt-drawing. It is possible to make tubular scaffolds with much smaller diameters. The PLC scaffolds are highly elastic in the circumferential direction as shown in Figure 3c. The radial elasticity exhibits great potential in TE of tubular tissues.

### 2.2. Customizability of Fiber Diameters and Tensile Properties

**Figure 4:**
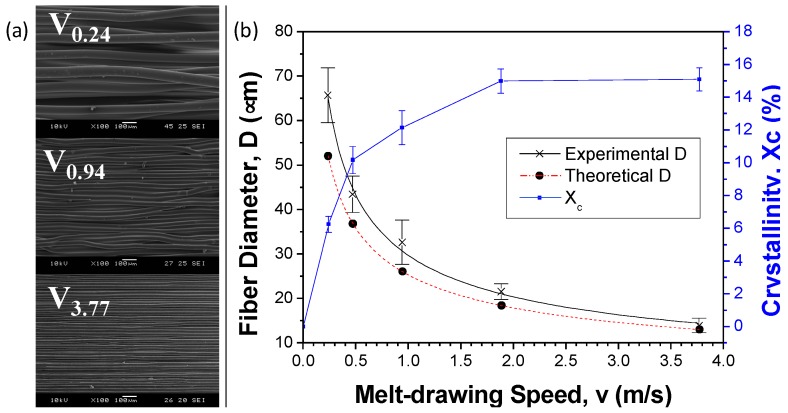
(**a**) SEM images of PLC scaffolds fabricated with different melt-drawing speeds; (**b**) Graphical illustration on the effect of melt-drawing speeds on the fiber diameters from theoretical and experimental. Effect of melt-drawing speeds on the crystallinity of PLC is included in the graph.

Figure 4a shows the surface morphology of the scaffold samples with different melt-drawing speeds. The highly aligned microfibers are clearly seen. The density of pristine PLC is 1.215 g/cm^3^ when computed using Archimedes method. Meanwhile the density percentage of the samples V_0.24_, V_0.47_, V_0.94_, V_1.88_ and V_3.77_ were determined to be 91.7%, 91.0%, 90.7%, 86.2%, 84.4%. The scaffolds’ porosity increases with the increasing melt-drawing speed. 

The fiber diameters of the samples V_0.24_, V_0.47_, V_0.94_, V_1.88_ and V_3.77_ were 65.7 ± 6.2, 43.5 ± 4.1, 32.6 ± 5.0, 21.5 ± 1.8, and 13.9 ± 1.7 µm, respectively. A model to empirically link the fiber diameter to the processing parameters was developed for the melt-drawing process [12]. The fiber diameter of PLC can be mathematically expressed in Equation (1) as:

(1) $$D={\left[\frac{\sqrt{2\text{gh}}}{v}\text{tanh}\left(\frac{\rho d\sqrt{2\text{gh}}}{12A}\text{exp}\left(\frac{-T_{g}{\left(T_{g}+150\right)}^{2}}{0.164T{\left(0.23T_{g}+150\right)}^{2}}\right)\right)\right]}^{\frac{1}{2}}$$

where h = 50 mm, ρ = 1.215 g/cm^3^, d = 2 mm, T = 423.2 K and T_g_ = 286.9 K [12] respectively. v represents the different melt-drawing speeds and A was calculated from Equations (2) and (3) to be 5.607 × 10^−5^ Pa·s when η = 1245 Pa·s at 150 °C.

(2) $$\eta =A\;\text{exp}\left(\frac{E_{a}}{\text{RT}}\right)$$

(3) $$E_{a}=\frac{{R\;T}_{g}{\left(T_{g}+150\right)}^{2}}{0.164{\left(0.23T_{g}+150\right)}^{2}}$$

The theoretical and experimental results of the fiber diameter were computed and shown in Figure 4b. The theoretical D was slightly underestimated but fit quite nicely to the experimental results. It demonstrates that the PLC fiber diameter can be tailored effectively by manipulating the melt-drawing parameters. Microfibers could be fabricated according to the desired fiber diameter in consequence of superb reproducibility and a uniform fiber distribution. Crystallinity of melt-drawn PLC was previously calculated [12] and plotted in Figure 4b. It is noted that the cast PLC was defined as zero melt-drawing speed in the results. A higher crystallinity is observed in the sample with a higher melt-drawing speed due to strain-induced crystallization.

**Figure 5:**
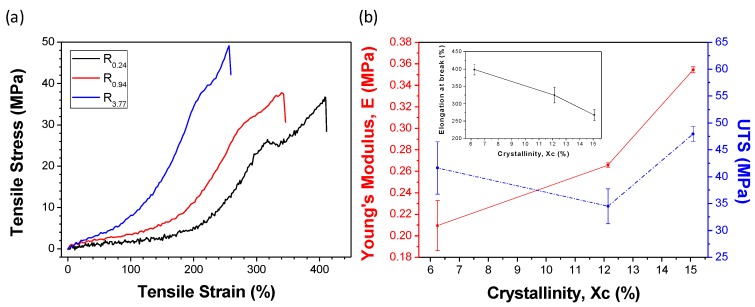
(**a**) Stress–strain curves for PLC rings with different melt-drawing speeds; (**b**) Relationship between Young’s modulus, UTS, and maximum elongation with crystallinity of PLC.

Mechanical properties of tubular organs differed from each other. For example, Young’s moduli of these tissues may vary from ~0.1 to ~4.4 MPa [42,43,44], ultimate tensile strength falls in the range of ~1.0 to ~4.5 MPa [43,44,45], and the maximum elongation can be up to ~200% [43,44,45,46]. The materials for TE can be chosen according to the mechanical properties needed. Here, tensile properties of PLC can be changed by varying the melt-drawing speeds to suit to some of the tubular tissues’ properties. The mechanical properties of the PLC are closely related to its crystallinity. Stress-strain curves of PLC rings R_0.24_, R_0.94_, and R_3.77_ are presented in Figure 5a. The deformation behavior of the highly elastic PLC ring is very similar to typical elastomeric materials [12]. Tensile properties of PLC are plotted against the crystallinity of the samples in Figure 5b. As expected, Young’s moduli (elasticity) and UTS have shown to be increase with increasing crystallinity. Elongation at break (ductility) of the samples decreases with the crystallinity % of PLC. 

### 2.3. Biocompatibility and MSC Seeding onto Aligned Microfibrous Scaffolds

**Figure 6:**
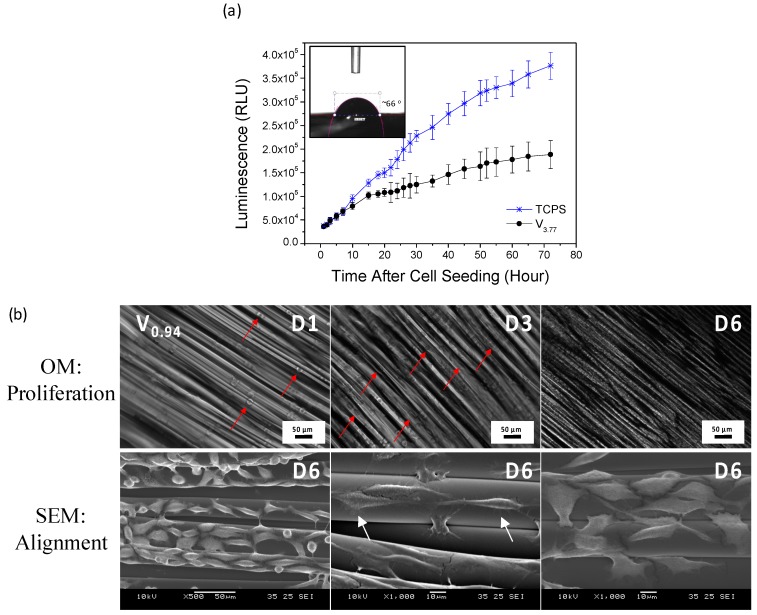
(**a**) Real-time cell proliferation of A10 on the TCPS (control) and the scaffold V_3.77_ with initial cell seeding of 2500 cells/scaffold. Inset: water contact angle of PLC scaffold; (**b**) OM images of L929 cell growth and distribution on V_0.94_ scaffold for day 1, 3 and 6. SEM images showing the L929 cells elongation and alignment on the scaffold after 6 days of culture.

As shown in Figure 6a, rat aorta A10 cells were proliferating well with time on the melt-drawn samples. The good biocompatibility is probably due to the low water contact angle of ~66° on the scaffold. There is no need to perform complex surface modification, such as surface grafting or ECM coating, for the cell attachment. Figure 6b exhibits that L929 cells growth and distribution is good on the scaffold surface. It is also shown that the L929 adhered well to the scaffold surfaces with a normal elongated spread morphology. Cells elongate by following the alignment of the microfibers. Agrawal et al. [41] also proved that smooth muscle cells aligned on the melt-drawn PCL fibers. Hence, the scaffold could provide 3D guided cell growth. 

After culturing hMSCs on the melt-drawn scaffolds for 5 days, the cells were immuno-stained for the myogenic protein, smooth muscle actin (SM actin). SM actin expressed in the cells indicative of differentiation along the SMC lineage. Although soluble factors, e.g., transforming growth factor β1 (TGF-β1) can induce mesenchymal stem cell (MSC) differentiation towards the smooth muscle cell (SMC) lineage, there are studies illustrating the potential to control stem cell fate via cell shape modulation and matrix elasticity [47,48,49]. Tay et al. [47] proved that enforced cell shape distortion results in physical impetus, by which the mechanical deformation is then translated into biochemical response. We proved that L929 cells were enforced to elongate following fiber direction. Meanwhile, according to Han et al. [48], MSCs cultured on a substrate with elasticity of around 1–500 kPa may express smooth muscle cell (SMC) markers. In this work, the scaffolds have elasticity ranging from 200 to 350 kPa, which might be the reason for SMC differentiation. 

**Figure 7:**
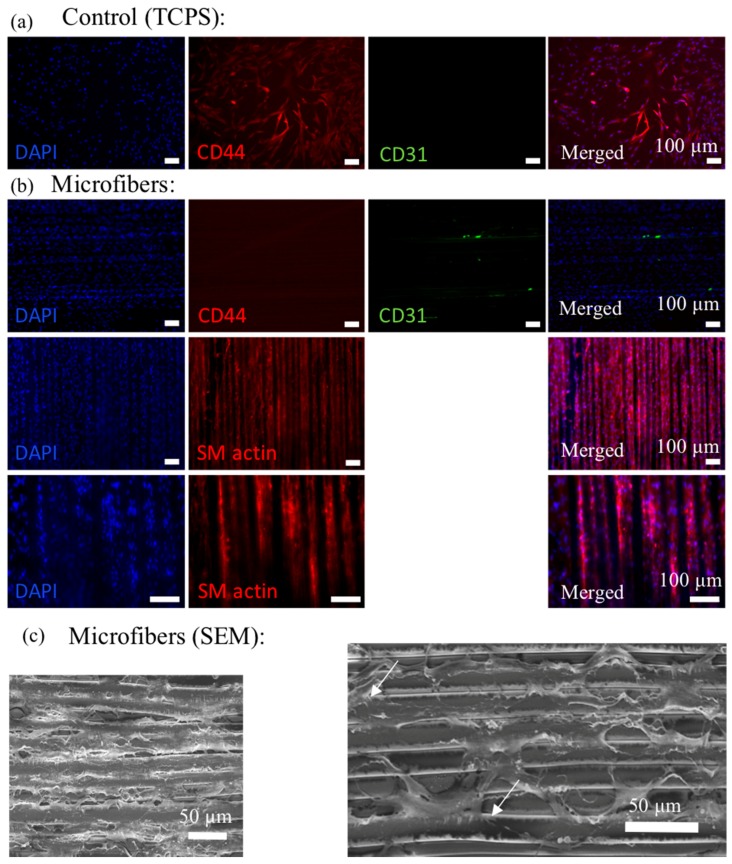
Cy3 labeled CD44 (red; positive marker for hMSCs), AlexaFluor 488 labeled CD31 (**green**; negative marker for hMSCs), DAPI nuclear staining (**blue**) and overlaid fluorescent image of immuno-stained cellular components (merged) for the hMSCs on control (TCPS) (**a**) and microfibers (**b**). Cy3 labeled SM actin (**red**), DAPI nuclear staining (**blue**) and overlaid fluorescent image of immuno-stained cellular components (merged) for the hMSCs on microfibers are shown in (**b**); (**c**) SEM images of the hMSCs on the microfibers show that cells can attach well on the microfibers.

The SEM micrograph of hMSCs cultured for 5 days on the scaffolds are shown in Figure 7c. Cells attached well on the microfibers, similar to the L929 cells attachment on the scaffolds. There are some filament-like focal adhesions structures that extend from these cell bodies and attach to the microfibers as illustrated in Figure 6b and Figure 7c (white arrow). These structures are expected to be adhesion sites between the cultured cells and the ECM [50].

### 2.4. Future Perspectives

The AM technique presented here shows great potential in producing personalized elastic scaffolds for different tubular organ TE. Dimensions of the scaffolds and the microfiber sizes can be varied by changing the fabrication parameters. Tensile properties of the scaffold also change with the melt-drawing speeds. In most of the native tubular tissues, such as the tunica media in blood vessels and the endocircular muscle layer in the esophagus, the smooth muscle tissues are organized in a concentric manner to provide mechanical strength and structural integrity to the tubes. Here, SMCs are cultured on the highly aligned fibers in the tubular direction which assist in cell alignment [41] and hence mimic the circumferential organization of SMCs in the native tubular organs. Furthermore, the hMSCs were able to differentiate to SMC lineage without any induction factors on these scaffolds. New smooth muscle tissues are gradually formed while the PLC scaffold degrades in eight months [51]. However, scaffolds should consist of highly porous structures with interconnected networks for nutrient and signaling molecule delivery, waste removal, and migration of cells [4,52]. Although the scaffolds fabricated here must have interconnected pores, the main drawback is that they have low porosities. Efforts are needed to increase the porosity of the scaffolds so that cell and tissue infiltration into the scaffolds can be enhanced. In addition, different biomaterials can be used to melt-draw the tubular scaffolds in order to expand their applicable range, e.g., desirable mechanical and degradation properties, for TE of different tubular tissues.

## 3. Materials and Methods 

### 3.1. Materials

PLC with l-lactide (LA) to ε-caprolactone (CL) with 70 to 30 molar ratio (Corbion Purac, Amsterdam, The Netherlands, PURASORB^®^ PLC 7015) is chosen in this work for the fabrication of tubular scaffolds. The copolymer has an inherent viscosity midpoint of 1.5 dL/g. Poly(l-lactide-co-ε-caprolactone) with a high LA ratio displays high deformation at break and high stiffness, while the copolymer with a high CL ratio possess an elastomeric thermoplastic behavior. PLC with LA:CL of 70:30 was chosen because it is sufficiently elastic while maintaining its high yield strength.

PLC degrades in 12 to 24 months according to the manufacturer. The slow degradation rate allows the PLC scaffold to maintain its mechanical strength before the tissue remodeling in vivo, which would eventually lead to a formation of normal tissue capable of self-support. On the other hand, in the PLC with LA:CL of 70:30, CL do not form crystallites [12]. Hence, the degradation time of the PLC is not too long, as compared to those copolymers that form CL crystallites where a foreign body is introduced to the body for a prolonged period of time. 

### 3.2. Rheological Characterization

Melt rheological characterization was performed on the TA DHR-2 rheometer (TA Instruments, New Castle, DE, USA) with dynamic oscillatory shear measurements. Viscosities of the PLC melt at different temperatures were monitored. Dynamic frequency sweep tests at 110, 120, 130, 140, and 150 °C were conducted with a parallel plate fixture of 25 mm in diameter at 2.0% strain and angular frequency from 0.1 to 100 rad/s. Zero-shear viscosity, η_0_ at low shear rates were plotted against the tested temperatures.

### 3.3. Fabrication of PLC Tubular Scaffolds

The melt-drawing device was employed to fabricate tubular scaffolds as described in our previous papers [11,12]. Briefly, the process involves melting the PLC at 150 °C in a melt holder and continuous drawing a single fiber from the melt. The microfiber was collected on a rotating cylindrical mandrel. The melt holder moved transversely along the length and distributed the microfibers on the mandrel layer by layer. The tubular scaffold formed as the microfibers merged which was then removed from the mandrel. 

Different dimensions of the mandrels were adopted in this work to show the size customization ability. The mandrels were designed by the means of computer aided design (CAD) and then 3D printed with PolyJet (Stratasys, Eden Prairie, MN, USA) [53]. To systematically study the effect of mandrel rotation speeds on the average fiber dimensions, a cylindrical mandrel with a diameter of 30 mm was adopted in this work. The mandrel rotational speeds of 150, 300, 600, 1200 and 2400 rotations per minute (RPM) were employed. These mandrel rotation speeds can be converted to the linear velocities (i.e., melt-drawing speeds) of 0.24, 0.47, 0.94, 1.88 and 3.77 m/s, respectively. The melt-drawing speeds are termed V_0.24_, V_0.47_, V_0.94_, V_1.88_ and V_3.77_ accordingly.

### 3.4. Gel Permeation Chromatography (GPC)

The molecular weight distribution of the pristine PLC and the melt-drawn PLC were studied to inspect the effect of melt-drawing on the polymer. The number-average molecular weights (M_n_) and the polydispersity indices (PDI) were measured at room temperature using GPC (Waters E2695 Alliance system with Waters 2414 RI detector, Milford, MA, USA). Chloroform was used as the effluent.

### 3.5. Scaffold Characterization

The surface morphology of microfiber scaffolds was examined under a scanning electron microscope (SEM, JEOL JSM-5600LV, Tokyo, Japan). The average microfiber diameter was computed from the SEM micrographs using the ImageJ software (National Institutes of Health (NIH), Bethesda, MD, USA). 

The Archimedes method was used to calculate the PLC density based on the Archimedes Principle. Weights of the pristine PLC and the melt-drawn PLC were measured in both air and ethanol at room temperature. Since the ethanol’s density is known at room temperature, the PLC samples’ densities can be computed.

Tensile properties were studied using melt-drawn PLC rings. The rings were each melt-drawn for 1 min, without moving the melt holder in transverse direction. Three different melt-drawing speeds (v = 0.24, 0.94, and 3.77 m/s) were adopted and the ring samples are named R_0.24_, R_0.94_, and R_3.77_, respectively. The samples have outer diameters of ~13.0 mm, ~21.0 mm, ~57.3 mm, respectively; and inner diameters of ~10.0 mm, ~17.9 mm, ~54.3 mm, respectively. The ring samples (*n* = 3) were mounted between two metallic arms, and then the uniaxial tensile tests were carried out with an Instron Universal Testing Machine (Model 5569, Norwood, MA, USA) with a load cell of 1 kN and a crosshead displacement of 20 mm/min. The ultimate tensile strengths (UTS), Young’s moduli, and elongation at break were computed from the results.

### 3.6. Biocompatibility Studies

A10 rat aorta myoblasts has been used in cell culture study to show the in vitro biocompatibility of the melt-drawn samples. A10 cells were cultured in the cell culture media of high glucose Dulbecco’s modified Eagle’s medium (DMEM; Gibco^®^, Thermo Fisher Scientific, Waltham, MA, USA) supplemented with 20% fetal bovine serum (FBS; Gibco^®^) and 1% antibiotic-antimycotic (Gibco^®^) in a humidified incubator (37 °C/5% CO_2_). Before cell seeding, the scaffolds were sterilized by immersion for 5 h in 70% ethanol solution, washed in a phosphate buffer solution (PBS; Sigma-Aldrich, St. Louis, MO, USA) and soaked in the correspondent culture medium for 1 h. The cells were detached from the culture flask upon confluency and seeded onto the scaffolds at a required cell density.

The A10 cell proliferation on the surface of the scaffolds was determined by the RealTime-Glo™ MT Cell Viability Assay (Promega, Fitchburg, WI, USA) to measure the cell viability in real time [12]. Cells were seeded on flat 5 × 5 mm^2^ microfibrous scaffolds at a density of 2500 cells/scaffold. The cellular constructs were maintained in the incubator at various time points for up to 72 h. The luminescence of the well content at several time points was measured using a microplate reader (Ultra Evolution, Tecan, Zürich, Switzerland). The luminescent signal is proportional to the number of viable cells in culture.

### 3.7. Cell Adhesion, Spreading and Alignment on Scaffolds

L929 murine connective tissue fibroblasts were cultured and seeded onto scaffolds, with the same conditions but in a DMEM media with 10% FBS. Cell adhesion, spreading and alignment of L929 cells were studied. Cells were seeded on flat 10 × 10 mm^2^ microfibrous scaffolds at a density of 1 × 10^5^ cells/scaffold. The constructs were maintained in an incubator and the cell growth was monitored daily using an inverted microscope (Zeiss, Axio Vert.A1, Oberkochen, Germany). The cell adhesion and alignment on the scaffolds was imaged using SEM after 6 days of culture. Before imaging, cellular constructs were rinsed with PBS and fixed with 3% glutaraldehyde overnight at 4 °C. Following PBS rinses, the samples were dehydrated through a series of graded alcohol solutions, and then dried using a critical point dryer (BAL-TEC, CPD 030, Los Angeles, CA, USA). The constructs were gold coated and observed under the SEM.

### 3.8. MSC Seeding onto Scaffolds

Human mesenchymal stem cells (hMSCs) from a 26-year-old female donor and cell culture medium were both obtained from Lonza (Basel, Switzerland). The hMSCs expressed CD 105/+, CD166/+, CD 44/+, CD 90/+, CD 73/+, CD 14/−, CD34/−, CD19/− and CD45/−. Information of the surface antigens were obtained via flow cytometry analysis provided in the company’s data sheet. hMSCs were expanded and cultured in mesenchymal stem cell basal medium according to the vendor’s instruction. For the cell seeding experiments, hMSCs were cultured in the same cell culture medium without using soluble differentiation factors. The cells were maintained at 37 °C in a humidified atmosphere of 5% CO_2_. Culture medium was changed every 2–3 days. 

hMSCs (passage 5) were seeded on flat 10 × 10 mm^2^ microfibrous scaffolds following the same protocol described above. Cells were seeded at a density of 2 × 10^5^ cells/scaffold. Culture medium was changed every 2–3 days.

### 3.9. Immunocytochemistry and Cell Adhesion Analysis

The hMSCs positive (CD44) and negative (CD31) markers, smooth muscle actin and cell nucleus were immuno-stained with staining kit (Thermo Fisher Scientific, Waltham, MA, USA) according to the manufacturer’s instructions. After 5 days of culture, cells were fixed with 4% paraformaldehyde for 15 min at room temperature. These cells were then permeabilized with 0.1% Triton X-100 in PBS for 10 min at room temperature and blocked with 5% bovine serum albumin (BSA) in PBS before the subcellular components were immuno-labeled. CD44, CD31 and cell nucleus were counter stained with rabbit monoclonal anti CD44 (2 μg/mL) counterstained with Cy3 goat anti rabbit IgG, mouse monoclonal anti CD31 (1:50) counterstained with AlexaFluor 488 donkey anti mouse IgG and 4′-6-Diamidino-2-phenylindole (DAPI) respectively. Smooth muscle actin (SMA) and cell nucleus were counter stained with rabbit monoclonal anti SMA (3 μg/mL) counterstained with Cy3 goat anti rabbit IgG, and DAPI respectively. Fluorescence images were visualized and imaged with Zeiss Axio Vert.A1. microscope. The cell adhesion on the scaffold was fixed, dehydrated and imaged using SEM after 5 days of culture as described above.

## 4. Conclusions

The tubular microfibrous PLC scaffold presented in this paper was fabricated using a simple but efficient method. Tubular scaffolds with various diameters, lengths and thicknesses were successfully fabricated. When melt-drawn at 150 °C, the fiber diameter can be altered by varying the melt-drawing speeds, conforming to the mathematical model developed. The scaffold fabrication is reproducible with macro- and micro-scale customizability. Increased strength but decreased elasticity and ductility of the PLC were proven to accompany the decrease in fiber diameter. In vitro biocompatibility evaluation showed that A10 and L929 attached and proliferated well on the PLC scaffolds. Cells were aligned by following the fiber alignment. hMSCs were successfully cultured on the microfibers, and induced expression of differentiation marker of SMC lineage in the absence of soluble induction media. This study suggests that melt-drawn PLC scaffolds would be a suitable substrate for potential applications in tubular organ TE, thanks to its customizability and biocompatibility.
